# Control of American Cockroach (*Periplaneta americana*) in Municipal Sewage Disposal System, Central Iran

**Published:** 2018-06-13

**Authors:** Ali Reza Zahraei-Ramazani, Abedin Saghafipour, Hassan Vatandoost

**Affiliations:** 1 Department of Medical Entomology and Vector Control, School of Public Health, Tehran University of Medical Sciences, Tehran, Iran; 2 Department of Public Health, Faculty of Health, Qom University of Medical Sciences, Qom, Iran; 3 Department of Chemical Pollutants and Pesticides, Institute for Environmental Research, Tehran University of Medical Sciences, Tehran, Iran

**Keywords:** *Periplaneta americana*, Control products, Sewage system, Iran

## Abstract

**Background:**

Cockroaches consists of 4000 species, of which 40 species are serious domestic pests. They have involved the mechanical transmission of various pathogenic viruses, bacteria, and protozoans to humans. This study aimed to determine different control methods of *P. americana* in sewage system of Esfahan City, central Iran using different insecticides recommended by WHO.

**Methods:**

Totally, 164 manholes with at least more than three cockroaches were selected until 2017 for this study. The species of cockroaches were identified; the adults and nymphs were counted and recorded. Each sewer shaft was assigned to one treatment method in an ascending order of number of cockroaches counted, coding the treatment method with paint on the lower part of the wall near the manhole cover. The manhole shafts were then inspected at 1 and 5 months’ post-treatment. Data analyzed by statistical methods.

**Results:**

Almost all of the products (Excluding boric acid with bait formulation) resulted in appropriate control within one month of application. The appropriate products for chemical control of cockroaches were the chlorpyrifos 5% Emulsifiable Concentrate (EC), diazinon 5% (EC), diazinon 0.05% (EC) and cypermethrin 5% Fog. These pesticides achieved an optimal reduction of population providing more than 90% control of cockroaches for five consecutive months.

**Conclusion:**

The emulsifiable concentrates and fog formulations in the control were more successful compared to other methods and this was penetration deep into the hiding places of cockroaches.

## Introduction

Cockroaches are one of the oldest inhabitants of earth, dating back as far as the Carboniferous period, over 250 million yr ago (1). They lack special adaptations like the sucking mouthparts in some insects such as the aphids and other true bugs (2). Over 4500 species of cockroaches have been identified, of which 40 species are associated with human habitants while four species are well known as pests (3–5).

Cockroaches have chewing mouthparts and feed on variety of materials (Omnivorous) aiding in the mechanical transmission of various pathogenic viruses, bacteria, and protozoans to humans (3, 4). Cockroaches have a worldwide distribution especially in the tropical and subtropical areas and can tolerate a wide range of environments from Arctic cold to tropical heat (3, 4). Their population increases in hot and humid places especially with the availability of food and water (6). Common indoor cockroaches found in Iran include, *Blattella germanica*, *Blatta orientalis*, *Periplaneta americana* and *Supella longipalpa* (3, 4).

*Periplaneta americana* is the largest species of cockroaches found in Iran. They originated from South America, measures 30–40 mm in length and are reddish brown in color. The American cockroach lives in hot areas of buildings like the kitchens, heating rooms, warehouses and sewage systems (4). They usually come out of their hiding places at night for feeding and other activities (7). The adult cockroaches are long-lived and can live for as long as one year or more producing large number of egg capsules during this period, depending on food availability (8).

Sewerage is established to collect and convey sewage in urban areas in order to prevent contamination of soil and water resources. Some species of cockroaches including *P. americana* have colonized the sewerages, turning them into suitable environment for reproduction and growth posing serious health problems to humans (9).

Two different methods are involved mainly in the control of cockroaches, chemical and non-chemical control methods. A third but not very common method of control involves the use of parasitoid wasp belonging to the family Evaniidae which naturally parasitize and destroy egg capsules of cockroaches. The non-chemical control method is not common, it involves the use of high-frequency ultra-vibration sound generating device. Chemical control method includes: residual spraying, dusting spray, mist spray and poisoned baits. The use of chemical insecticides to control cockroaches is the most popular method. Fire and fogging have been proposed also in the control of *P. americana* in sewers, steam tunnels, storehouses and outdoor areas (10, 11).

The main pesticides used in chemical control of cockroaches are classified into three classes, pyrethroids, carbamates and organophosphorus. Organochlorine is also used in some countries (12).

In recent years, the extensive use of pesticides in the control of cockroaches has led to the development of resistance making cockroaches to be ranked second after house flies (*Musca domestica*) with regards to insecticides resistance (13). Dichlorodiphenyltrichloroethane (DDT) is widely used in the control of cockroaches across some Countries, but in America, it was replaced with chlordane which is more effective. Chlordane, diazinon and malathion are three insecticides widely used in America to control cockroaches, while in Great Britain, diazinon and dieldrin are used often (14).

Although, many researchers to achieve appropriate and effective insecticides control against cockroaches using newer methods are still ongoing (15). The determination of an appropriate and effective insecticide for the control of *P. americana* in sewage system of Esfahan is necessary, due to the high population of this species as a result of hot and humid environment suitable for cockroaches. Resistance of cockroaches to insecticides can be delayed or avoided by using an appropriate insecticide. Different chemical products and physical control method were studied in three locations with old sewerage collection networks at Esfahan City until 2017 for the control of American cockroach.

## Materials and Methods

### Study area

Esfahan is the capital city of Esfahan Province with an area of 250km^2^ and 1695789 residents. It is the second most populous metropolitan city after Tehran in the central of Iran. Its altitude is 1571m, latitude 32°38′30″N and longitude 51°39′40″E. This city is located in a dry semi-desert region with an annual rainfall of 102mm, average summer maximum and winter minimum temperatures of 37.1 °C and −5.81 °C, respectively (16).

**Fig. 1.**
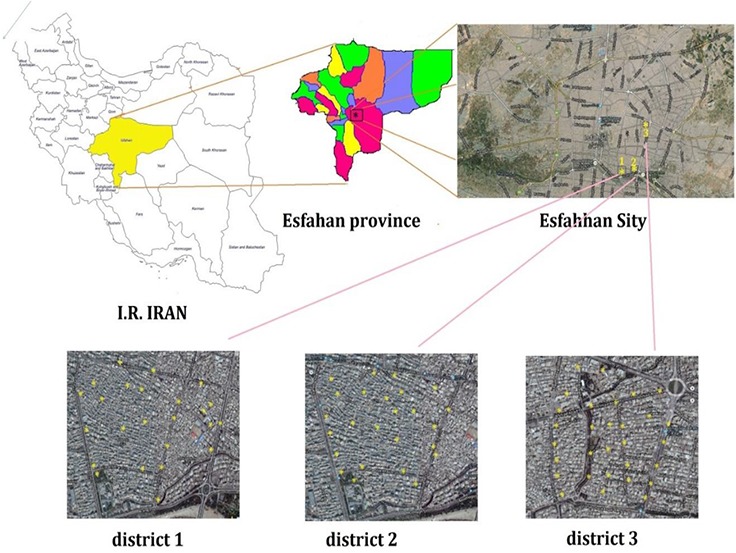
Location of Esfahan Province and city located in central part of Iran

The study was conducted in three areas with old sewer networks with permission from Esfahan’s Water and Wastewater Company (Fig. 1). Esfahan City has over 55375 manholes in its sewage system with an average depth of 9m on the main lines and 1meter on lateral lines. Most of the manhole covers were cast irons with two holes, and the average diameter of the holes was three centimeters. The shafts of the manholes were made of brick and cement coverings, and their stables moderate warmth and high humidity which provide excellent conditions for cockroaches.

Totally, 164 manholes with at least more than three cockroaches were selected until 2017. The cast-iron covers were removed from each manhole and the species of cockroach infesting the shaft were noted, the adults and nymphs were counted and recorded. Strong flashlights were used to facilitate counting, shafts with more than 300 cockroaches were estimated by counting 0.25m^2^ section of the shaft and multiplying by the number of sections infested. Each sewer shaft was assigned to one treatment method in an ascending order of number of cockroaches counted, coding the treatment method with paint on the lower part of the wall near the manhole cover. The manhole shafts were then inspected at 1 and 5 months after treatment.

### Control products

The insecticide formulations used include, (1) Aqueous sprays of 5% (WP) carbaryl, 5% (WP) diazinon, 5%, 0.5%, 0.05% (EC) diazinon, 5%, 0.5%, 0.05% (EC) chlorpyrifos, and 0.02% (SC) responsar Beta-cyfluthrin (2). Thermal fogs 0.028g/m^3^ of cymperator (synthetic pyrethroid), cypermethrin and tetramethrin (EC), diazinon 0.5g/m^3^, and chlorpyrifos 0.26g/m^3^. (3) Baits of 5% carbaryl and 50% boric acid. All the insecticides were prepared according to the description (17). All of applied pesticides in this study were purchased from Bayer Company (Bayer Persian AG), Tehran, Iran. Fire with oil (10g/m^2^) was used to burn 10 manhole shafts and twenty untreated infested manholes were used for control too. Number of cockroaches per manhole was counted monthly for 5 months’ post-treatment. Aqueous sprays were applied with HUDSON X-Pert®110 power compression sprayer equipped with a nozzle, flexible hose, stainless steel tubular spray tip extension on the handle grip, delivering 757cm^3^/ minute. The extension tube was lowered to the bottom of the shaft and the tip was raised upwards at a uniform rate of 0.5m/sec while the spray was applied. A 5% spray, therefore delivered about two grams of the active ingredient/m^2^. Boric acid baits in Petri dishes were placed in vases in dry ledge at the bottom of the shaft. Thermal fog with solutions was applied from a “Light-weight Model” TF 30. The TF 30 was operated at a combustion chamber temperature of 600 °C, a dial flow setting of four l/h. Each manhole was treated for five seconds per m^3^. Fire was applied with HODSON X-Pert®110 filled with oil, the extension tube was lowered almost to the bottom of the shaft and spray was applied raising the tip upwards at a uniform rate of 10g/m^2^. Oxygen also was blown into the manhole with an electrical fan equipped with a flexible hose.

The study was approved and carried out under the guidelines of Ethical Committee of the School of Public Health, Tehran University of Medical Sciences, Tehran, Iran. The study was conducted on sewerage system of Esfahan City with permission obtained from the General Directorate of Environment.

### Statistical analysis

Efficacy was calculated from the visual counts of cockroaches in the manholes made periodically for up to five months after treatment. The total numbers of cockroaches per treatment were grouped. For any given treatment, Data analysis was done using SPSS software (Chicago, IL, USA) and a repeated measures ANOVA design was used to determine different control methods of *P. americana* in sewage system.

## Results

The effect of 15 types of pesticides or control products on American cockroaches was investigated. The number of examined manholes was 164. Twenty manholes were used as controls. Table 1 showed the mean and standard deviation of American cockroach’s frequency prior, one month and five months after intervention.

**Table 1.** The mean and standard deviation of American cockroach’s frequency at prior one, one month and five months after the intervention, Esfahan City, Iran

**Control products**	**Number of manholes**	**Formulation**	**Pre treatment**		**After 1 month**		**After 5 month**	

			**Mean**	**SD**	**Mean**	**SD**	**Mean**	**SD**
**Carbary l5%**	10	WP	66.50	4.007	1.50	.972	66.20	4.872
**Diazinin 5%**	10	WP	58.40	3.098	0.00	0.00	27.60	2.271
**Chlorpyrifos 5%**	12	EC	25.42	2.065	0.00	0.00	0.00	0.00
**Chlorpyrifos 5%**	10	WP	59.90	4.606	0.00	0.00	5.70	1.889
**Chlorpyrifos 0.05%**	10	EC	62.90	2.923	0.00	0.00	8.30	1.567
**Diazinon 5%**	13	EC	119.00	6.232	3.54	.877	1.00	0.707
**Diazinin 0.5%**	10	EC	41.40	2.797	0.10	0.316	6.70	1.418
**Diazinon 0.05%**	5	EC	37.60	3.209	0.60	0.548	5.00	1.000
**Responsar 0.02%**	10	SC	38.40	2.914	0.0	0.0	8.50	0.972
**Cympermethrin**	10	Fog	63.40	2.066	0.00	0.00	3.70	1.059
**Diazinon**	10	Fog	103.70	4.572	0.00	0.00	7.50	1.780
**Chlorpyrifos**	10	Fog	82.00	5.354	0.00	0.00	.10	0.316
**Boric Acid**	5	Bait	22.20	2.490	0.80	0.447	3.40	0.894
**Carbary l5%**	9	Bait	38.33	2.646	1.00	0.707	5.89	1.537
**Fire**	10	-	61.30	2.406	0.20	0.422	9.80	2.201
**Untreated**	20	-	41.05	3.017	19.65	2.254	40.60	3.393
**Total**	164	-	59.12	26.591	2.88	6.402	14.40	18.444

The results of tests of Between-Subjects Effects showed that there was a significant difference between the frequency of adult cockroaches prior, one month and five months after intervention (F= 515.96, P< 0.001). Post hoc test showed that most of the pesticides and other control products showed significant differences (Table 1).

Table 2 shows the mean difference of American cockroach’s frequency prior, one month and five months after the intervention compared to untreated. The highest mean difference of adult cockroaches was related to chlorpyrifos 5% (25.29) and then Boric Acid (24.97. There was a significant difference between Mean Difference of all tested pesticides and control (P< 0.05).

**Table 2.** Mean Difference of American cockroach’s frequency at before, one month and five months after the intervention compared to untreated, Esfahan City, Iran

**Control products**	**Mean Difference**	**95% Confidence Interval**	

		**Lower limit**	**Upper limit**
**Carbary l5%**	10.97	9.22	12.72
**Diazinin 5%**	−5.10	−6.85	−3.35
**Chlorpyrifos 5%**	−25.29	−26.94	−23.65
**Chlorpyrifos 5%**	−11.90	−13.65	−10.15
**Chlorpyrifos 0.05%**	−10.03	−11.78	−8.28
**Diazinon 5%**	7.41	5.80	9.02
**Diazinin 0.5%**	−17.70	−19.45	−15.95
**Diazinon 0.05%**	−19.37	−21.62	−17.11
**Responsar 0.02%**	−18.13	−19.88	−16.38
**Cympermethrin**	−11.40	−13.15	−9.65
**Diazinon**	3.30	1.55	5.05
**Chlorpyrifos**	−6.40	−8.15	−4.65
**Boric Acid**	−24.97	−27.22	−22.71
**Carbary l5%**	−18.69	−20.51	−16.88
**Fire**	−10.0	−11.75	−8.25

## Discussion

American cockroach is an urban pest that is prevalent in sewerage collection networks in tropical and subtropical countries. In this study, *P. americana* was the most prevalent species in Esfahan sewerage system. The finding is in agreement with the results of previous studies (18, 19). *Periplaneta americana* is commonly found in urban regions that have sewage pipe-lines connected to human dwellings, which is suitable for this species growth, reproduction, and distribution (7).

Almost all the chemical products (Except boric acid with bait formulation) and fire used in this study resulted appropriate for control of cockroach at the end of the first month. Efficacy and persistence of insecticides in old sewer systems depend on several factors such as climatic conditions, amounts of organic matter, the population of cockroaches, the presence of resistant population, the type of active matter and the kind of formulation. There were many problems on the control of cockroaches with the use of pesticides for several reasons (20), and the main reason is that commonly used insecticides may lead to resistant in cockroaches. Besides, many pesticides are repellents to cockroaches and their usage is avoided.

Chemical control methods provide only temporary control, it should be accompanied by other control methods such as solid waste management, water and wastewater treatment, industrial waste treatment, noise, pollution control and house improvement (20). The use of chemical control method to reduce population of cockroaches is limited by several factors such as the development of natural resistance in *P. americana* population and its negative impacts on human health. *Periplaneta americana* takes between 4–12 months to complete its life cycle and the number of generations per year is not as many as in other species, therefore the expression of resistant genes could take more time (21).

The use of pesticides with strong vector control management is the most efficient method of controlling this pest in many regions of the world. Various formulations (sprays, dust and baits registered) have been used in the control of *P. americana*, but there is little evidence as to their efficacy in sewers. Aqueous sprays of wettable powder of carbaryl and diazinon provides excellent result in the control of *P. americana* population at least for one month. However, the effects of these pesticides on the cockroaches lasted for less than five months. This was due to the inability of the pesticides to penetrate the egg sacs (Oothecae) and the long life cycle and breeding of cockroaches in the winter. Some insecticides lose their effectiveness after one month of application, as we observed the population of cockroaches after 5 months did not decrease but increased. Chlorpyrifos 5% EC, diazinon 5% EC and diazinon 0.05% EC were suitable pesticides for cockroach control which provided an optimal reduction of *P. americana* populations for five months respectively. These pesticides provide more than 90% control for five months. Spraying emulsion with chlorpyrifos and diazinon in water (EC) provides a quick, temporary knockdown of cockroaches, they give long-term control relatively.

Sprays are highly repellant to cockroaches and should not be applied to surfaces in areas where traps or bait stations are located. Sprays can also disperse cockroaches to other areas of the manhole from which they could later return. Moreover, cockroaches have become resistant to many insecticides that formerly were used to control them. For example, recently, the American cockroach has been found to be resistant to trichlorfon and diazinon in China USA (15). The results of this present study indicate chlorpyrifos 5%, diazinon and cymperator 5% used as fog formulation provides an excellent reduction of American cockroaches at the end of one and five months. At the end of the first month of treatment, 100% cockroach reduction was achieved and 91%, 81.7% and 99% at five months using these three pesticides.

Thermal fogging was observed to be the best and simplest method for cockroaches control in sewer system of Esfahan. These methods were successful and consistent because thermal fogs of these pesticides penetrated deeply into the hiding places and they were particularly useful in basements of buildings, sewers and drainage systems. This method in the winter provides 99% reduction for up to five months and if used twice in a year, the cockroach’s population in the manholes will be decreased to zero. This technique works in control program of cockroach; it requires specialized equipment. Baits of carbaryl 5% and boric acid were used and they provided short time (one month) control of cockroaches in sewage system (98.3% and 83.9%). Their effects reduced over time and the methods were unsuccessful consistently because the cockroaches quickly consumed all the baits in the shafts and it was difficult and time-consuming to replace them.

An important factor reported to affect baits performance against American cockroaches is sanitation (22). Sanitation has been shown not to have a direct relationship with the level of domiciliary cockroach infestation in Malaysia (23) (L) was investigated in residential and clustered setting according to their sanitary conditions, results indicate that at 1-week post-treatment, houses with good sanitary conditions showed significantly and faster reduction in the number of cockroaches trapped (> 95%) than those with moderate and poor conditions. In this study the control effect of Responsar 0.02% as suspension concentrate formulation at the end of one month was 100% but at the end of 5 months decreased to 71%. The use of residual insecticides to control cockroaches when their growth is slow (In winter) have a long-term effect in the control of cockroaches in sewerage systems. Use of fire with oil (10g/m^3^) as a physical control method was applied in 10 manholes and caused 99.7% and 76% reduction of *P. americana* after one and five months respectively.

## Conclusion

The use of fire in manholes of sewerage systems is a suitable strategy in the control of cockroaches if done using proper equipment because it destroys the oothecae of cockroaches too. The advantage of these strategies was that lateral lines and adjacent manholes could be treated using these pesticides. Moreover, these control methods require specialized equipment. Chemical control methods are a common method of *P. americana* control around the World but it has been limited by several factors: the development of natural resistance and the chemical insecticides are expensive, toxic to humans and other living beings, and they are harmful to the environment.
